# Supplementary material for: Rescue Potential of Supportive Embryo Culture Conditions on Bovine Embryos Derived from Metabolically Compromised Oocytes
Source: Int J Mol Sci. 2020 Nov 2;21(21):8206. doi: 10.3390/ijms21218206 (PMC7663530; doi:10.3390/ijms21218206)
Supplement: Supplementary file 1 [file ijms-21-08206-s001.pdf]

Rescue potential of supportive embryo culture conditions on bovine embryos derived from metabolically-compromised oocytes

Supplementary Materials:

Table S1. The effect of ITS or serum supplementation during IVC on developmental competence within the same *in vitro* maturation group (BASAL, PA and HCOMBI):

Maturation	Culture	Oocytes used	Cleaved embryos	Two-cell stage	Four-cell plus	Fragmented embryos	Day 7 blastocysts	Day 8 blastocysts
**Basal**	BSA	515	395^{ab} (76.69%)	48^{a} (9.32%)	255^{a} (49.51%)	63 (12.23%)	105 (20.38%)	125 (24.27%)
Basal	ITS	510	406^{a} (79.6%)	63^{ab} (12.35%)	250^{a} (49.01%)	56 (10.98%)	124 (24.31%)	139 (27.25%)
Basal	Serum	488	348^{b} (71.31%)	73^{b} (14.95%)	182^{b} (37.29%)	55 (11.27%)	119 (24.39%)	136 (27.86%)
**PA**	BSA	270	176 (65.18%)	42 (15.55%)	81$^{xy\$\S}$ (30.00%)	35 (12.96%)	30^{x} (11.11%)	37^{x} (13.70%)
PA	ITS	278	200§ (71.94%)	43 (15.46%)	110$^{x\$}$ (39.56%)	30 (10.79%)	52^{y} (18.7%)	68^{y} (24.46%)
PA	Serum	247	156§ (63.15%)	54 (21.86%)	53^{y§} (21.45%)	30 (12.14%)	30^{xy} (12.14%)	45^{xy} (18.21%)
**HCOMBI**	BSA	527	377 (71.53%)	63 (11.95%)	221 (41.93%)	65 (12.33%)	87^{j} (16.50%)	96§ (18.21%)
HCOMBI	ITS	525	387 (73.71%)	46 (8.76%)	235 (44.76%)	84§ (16.00%)	101^{jk} (19.23%)	120 (22.85%)
HCOMBI	Serum	377	275 (72.94%)	55 (14.58%)	149 (39.52%)	41§ (10.87%)	88^{k} (23.34%)	91§ (24.13%)

% values refer to proportions from total number of used oocytes. Values with different superscripts within the same *in vitro* **maturation** group are significantly different at $P<0.05$. Values labeled with a, b are used within BASAL IVM; x, y is used for maturation in the presence of PA; j, k is used within HCOMBI IVM. Values labeled with "§" or "$" tend to be different from each other at $P<0.1$ and >0.05.

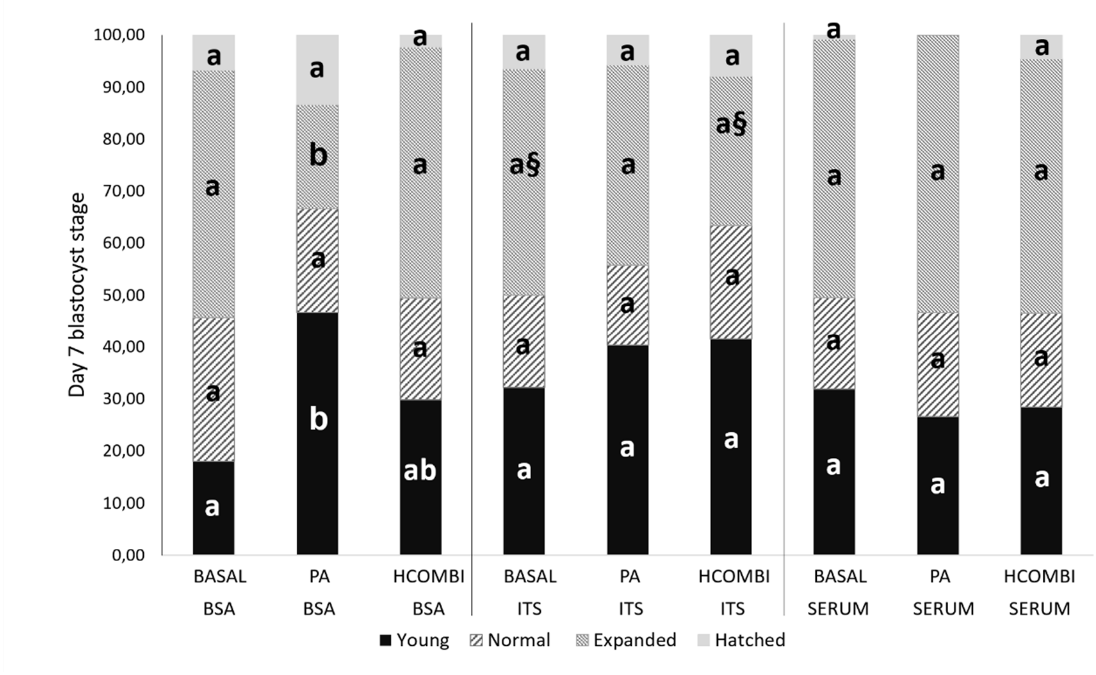

Figure S1. Proportions (%) of different stages of blastocysts resulting from oocytes exposed to BASAL, PA and HCOMBI NEFAs during maturation and cultured in the presence of absence of BSA, ITS or serum. Values with different superscripts per embryo stage within the same *in vitro* **culture** group are significantly different at P<0.05. Values labeled with "§" tend to be different from each other at P<0.1 and >0.05.

Table S2a. The effect of ITS or serum supplementation during IVC on mRNA expression (fold change) of genes related to cellular stress of blastocysts within the same *in vitro* maturation group (BASAL, PA and HCOMBI). Data are presented as mean fold change ± SEM.

Matur-ation	Culture	HSP60 fold change	GPx fold change	Catalase fold change	ATF~~tf~~6 fold change	TFAM fold change	HSP10 fold change	SOD2 fold change	ATF~~tf~~4 fold change
**BASAL**	BSA	1	1^{a}	1	1	1	1	1	1
BASAL	ITS	1.37±0.47	0.78±0.16^{b}	1.26±0.37	1.07±0.21	2.00±0.64	1.95±0.85	1.16±0.69	1.02±0.20
BASAL	Serum	1.43±0.40	0.42±0.06^{b}	1.61±0.39	1.57±0.64	1.63±0.56	1.88±0.66	1.56±0.78	0.80±0.12
**PA**	BSA	1.88±0.35^{xy§}	0.79±0.11^{x}	1.18±0.17	1.15±0.31	0.98±0.16^{xy§}	1.44±0.4	1.18±0.33	0.82±0.24
PA	ITS	1.05±0.14^{x§}	1.03±0.09^{x}	0.98±0.06	0.90±0.15	1.40±0.12^{x§}	1.27±0.10	1.09±0.09	0.91±0.19
PA	Serum	1.50±0.06^{y}	0.31±0.02^{y}	0.81±0.08	0.77±0.08	0.99±0.09^{y}	1.03±0.07	0.80±0.04	0.60±0.08
**HCOMBI**	BSA	1.06±0.23	1.00±0.09^{j}	1.18±0.34	1.32±0.39	1.32±0.38	1.45±0.35	1.46±0.53	1.28±0.29
HCOMBI	ITS	0.78±0.10	1.03±0.15^{j}	0.96±0.16	1.11±0.28	1.29±0.22	1.15±0.12	0.97±0.14	1.02±0.05
HCOMBI	Serum	1.20±0.14	0.34±0.09^{k}	1.32±0.06	2.03±0.60	1.05±0.11	1.61±0.37	0.95±0.10	0.89±0.14

Values with different superscripts within the same *in vitro* **maturation** group are significantly different at P<0.05. Values labeled with a, b are used within BASAL IVM; x, y is used for maturation in the

presence of PA; j, k is used within HCOMBI IVM. Values labeled with "§" or "$" tend to be different from each other at P<0.1 and >0.05.

Table S2b. mRNA expression (fold change) of genes related to cellular stress of blastocysts resulting from BASAL, PA or HCOMBI exposed oocytes cultured in serum, BSA and/or ITS. Data are presented as mean fold change ± SEM.

**Matur-ation**	**Culture**	**HSP60 fold change**	**GPx fold change**	**Catalase fold change**	**ATF~~tf~~6 fold change**	**TFAM fold change**	**HSP10 fold change**	**SOD2 fold change**	**ATF~~tf~~4 fold change**
BASAL	BSA	1[a]	1	1	1	1	1	1	1
PA	BSA	1.88±0.35[b§]	0.79±0.11	1.18±0.17	1.15±0.31	0.98±0.16	1.44±0.41	1.18±0.33	0.82±0.24
HCOMBI	BSA	1.06±0.23[ab§]	1.00±0.09	1.18±0.34	1.32±0.39	1.32±0.38	1.45±0.35	1.46±0.53	1.28±0.29
BASAL	**ITS**	1.37±0.47	0.78±0.16	1.26±0.37	1.07±0.21	2.00±0.64	1.95±0.85	1.16±0.69	1.02±0.20
PA	ITS	1.05±0.14	1.03±0.09	0.98±0.06	0.90±0.15	1.40±0.12	1.27±0.10	1.09±0.09	0.91±0.19
HCOMBI	ITS	0.78±0.10	1.03±0.15	0.96±0.16	1.11±0.28	1.29±0.22	1.15±0.12	0.97±0.14	1.02±0.05
BASAL	**Serum**	1.43±0.40	0.42±0.06	1.61±0.39 [jk§]	1.57±0.64 [jk]	1.63±0.56	1.88±0.66	1.56±0.78	0.80±0.12
PA	Serum	1.50±0.06	0.31±0.02	0.81±0.08 [j§]	0.77±0.08 [j]	0.99±0.09	1.03±0.07	0.80±0.04	0.60±0.08
HCOMBI	Serum	1.20±0.14	0.34±0.09	1.32±0.06 [k]	2.03±0.60 [k]	1.05±0.11	1.61±0.37	0.95±0.10	0.89±0.14

Values with different superscripts within the same *in vitro* **culture** group and column are significantly different at $P < 0.05$. Values labeled with a and b are used within BSA IVC; x is used for culture in the presence of ITS; j and k are used within Serum IVC. Values labeled with "§" or "$" tend to be different from each other at $P < 0.1$ and >0.05.

Table S3. The effect of 0.5% ethanol (Solvent) or BASAL NEFA concentrations during IVM on developmental competence:

Maturation	Culture	Oocytes used	Cleaved embryos	Day 8 blastocysts
Control	BSA	84	63 (72.9%)	25 (30.4%)
Solvent	BSA	79	51 (63.9%)	21 (26.6%)
BASAL	BSA	84	59 (71.2%)	21 (25.1%)

% values refer to proportions from total number of the used oocytes. None of the displayed differences were significant (P>0.1).

Table S4. Details of primers used for qPCR

Gene	Forward primer (5′→3′)	Reverse primer (5′→3′)	Product size (bp)	Gene Bank accession number
SOD2	TGCAAGGAACAACAGGTCTTATC	CTCAGTGTAAGGCTGACGGTT	181	NM_201527.2
GPX1	AACGTAGCATCGCTCTGAGG	TCTCCTCGTTCTTGGCGTTT	145	NM_174076.3
CAT	CTATCCTGACACTCACCGCC	GAAAGTCCGCACCTGAGTGA	268	NM_001035386.2
HSP10	CAACGGTGGTAGCTGTTGGA	TGGTGCCTCCATATTCTGGG	108	NM_174346.2
HSP60	CTACTGTACTGGCACGCTCT	CAATCTCTTCGGGGGTTGTC	159	NM_001166608.1
ATF4	TTTCTGAGCAGCGAGGTGTT	AAGCATCCTCCTTGCTGTTG	214	NM_00134341.2
ATF6	CGAGGATGGGTTCATAGGCA	GACGGCCATGAGCTGAGAAT	126	BC120388.1
TFAM	GCCAAGCTATGGAGGGAACT	AGCTTTACCTGTGATGTGCCA	293	NM_001034016.2
18S	AGAAACGGCTACCACATCCA	CACCAGACTTGCCCTCCA	169	AF176811.1
YWHAZ	GCATCCCACAGACTATTTCC	GCAAAGACAATGACAGACCA	120	BM446307.1
H2A	CGGAATTCGAAATGGCTGGC	TCTTTCGATGCATTTCCTGCC	238	NM_174809.2
BAX	AGCAGATCATGAAGACAGGG	TCAGACACTCGCTCAGCTTC	141	NM_173894.1
